# Data on U.S. state-level electric vehicle policies, 2010–2015

**DOI:** 10.1016/j.dib.2019.01.006

**Published:** 2019-01-08

**Authors:** Sherilyn Wee, Makena Coffman, Sumner La Croix

**Affiliations:** a University of Hawaii Public Policy Center and University of Hawaii Economic Research Organization (UHERO), University of Hawai‘i at Mānoa, United States; b Department of Urban and Regional Planning and UHERO, University of Hawai‘i at Mānoa, United States; c Department of Economics and UHERO, University of Hawai‘i at Mānoa, United States

**Keywords:** AFDC, Alternative Fuel Database Center, BEV, battery electric vehicle, EV, electric vehicle, CPI, consumer price index, EREV, extended range electric vehicle, HOV, high occupancy vehicle, MSRP, manufacturer suggested retail prices, PHEV, plug-in hybrid vehicle, TTI, Texas A&M Transportation Institute, TOU, time-of-use rates, VLT, vehicle license tax, Electric vehicles, State policies

## Abstract

This data set documents the duration and value of U.S. state and local electric vehicle (EV) policies in effect from 2010 to 2015. Though the focus is on policies at the state-level, local government and electric utility policies are documented when they collectively cover a majority of the state׳s population or electricity customers. Data were collected first from the Alternative Fuel Database Center (AFDC), then supplemented with information taken from more than 300 government (state, city, and county) and utility websites. Nine separate EV-related policy instruments were identified, organized as capital financial incentives, operating financial incentives, preferred access incentives, and disincentives. Though most policy instruments act to support EV adoption, an increasing number of U.S. states are adopting an annual fee for EVs to support road maintenance costs. For *vehicle purchase incentives*, *home charger subsidies*, *vehicle license tax* or *registration fees*, and the *annual EV fee,* data was gathered on the money value of these policy instruments. For *emissions inspection exemptions* and *high occupancy vehicle (HOV) lane access*, an annual money value for each policy instrument is estimated. The other policy instruments, *time-of-use (TOU) rates* for electricity, *designated parking* and *free parking*, are reported as binary variables. For further discussion of EV policy instruments as well as interpretation of their values, see Wee et al. [1]. EV policy instruments often differentiate between all-battery electric vehicles (BEVs) or plug-in hybrid electric vehicles (PHEVs). Data is similarly organized with this distinction.

**Specifications table**

**Table:** 

Subject area	*Public Policy, Transportation*
More specific subject area	*Alternative fuel vehicles, electric vehicles, policy analysis*
Type of data	*Tables and figures*
How data was acquired	*Alternative Fuels Database Center (AFDC); government websites; power utility websites; follow-up phone calls and emails with relevant agencies/organizations*
Data format	*Raw, analyzed and estimated.*
Experimental factors	*Data was collected by semi-annual period by model.*
Experimental features	*Qualitative data analysis.*
Data source location	*All data series are for the 50 U.S. states.*
Data accessibility	*Data are provided in this article.*

**Value of the data**

• A comprehensive database for all consumer-oriented state and some local government policies affecting EV adoption in the United States is provided.
• Nine distinct policy instruments that states, counties, cities, and utilities use to incent EV adoption is identified.
• A growing number of states have enacted an additional annual fee for registering an EV rather than a gasoline-powered vehicle.
• This dataset is valuable for researchers interested in analysis of the effects of public policies on adoption and use of EVs.

## 1 Data

**Fig. 1:**
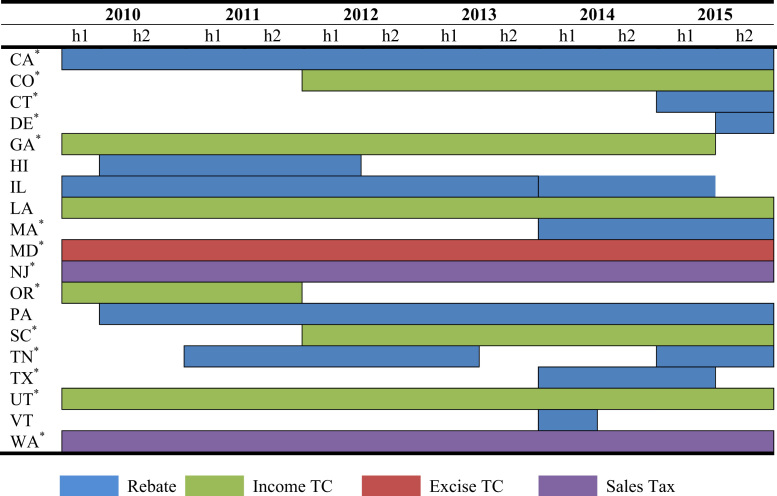
Timing and type of EV purchase incentives. Note: TC stands for Tax Credit ^*^Leased vehicles are also eligible for the purchase incentive. Leased vehicles were eligible since 2014h2 in MD, 2015h1 in TN, and 2015h1 in Utah.

**Fig. 2:**
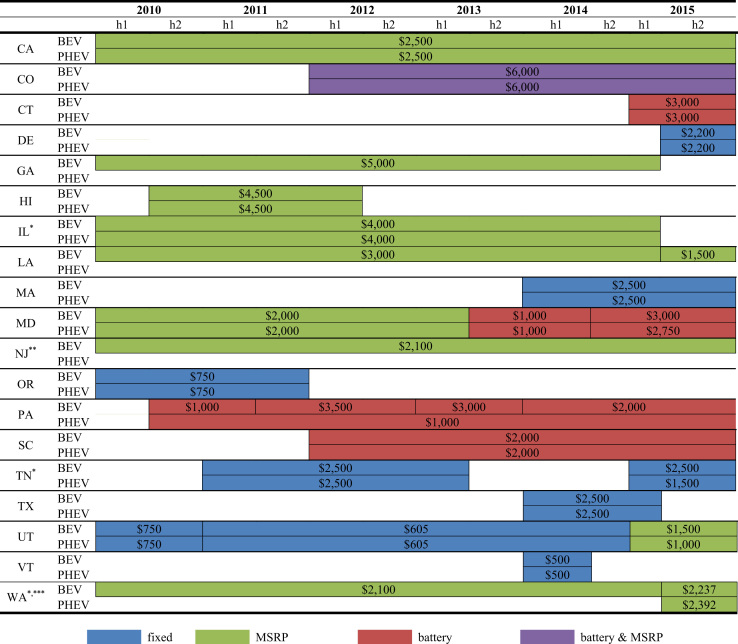
Maximum value of EV purchase incentives. ^*^Subsidy for PHEVs only applies to Extended Range Electric Vehicles (EREVs). In TN, this was the case for the first round of subsidies (2011h1 - 2012h2). ^**^Based on an average MSRP of $30,000 for the Nissan Leaf and 7 percent sales tax. ^***^Assumes a minimum 7 percent sales tax (varies by county). For illustration, the BEV amount between 2010h1 and 2015h1 is 7 percent of $30,000. In 2015h2, the sales tax exemption expanded to include EREVs, but restricted eligible vehicles (both BEVs and EREVs) to those valued at under $35,000. As a result, the only eligible EREV is the Chevrolet Volt. Based on the vehicles that were actually sold in 2015h2, the maximum BEV amount corresponds to a vehicle in the price range of a Kia Soul EV.

**Fig. 3:**
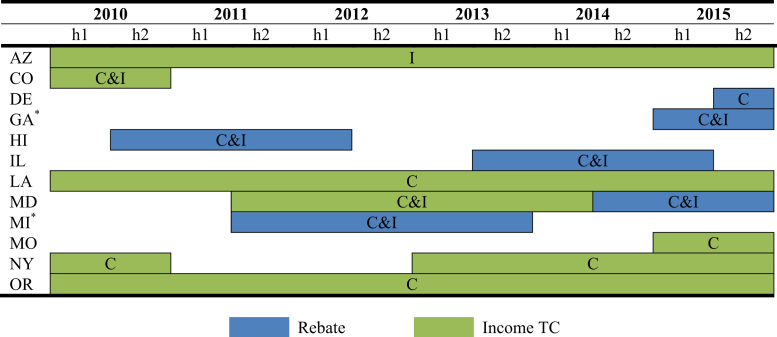
Timing and type of home charger subsidy. ^*^Utility incentive.

**Fig. 4:**
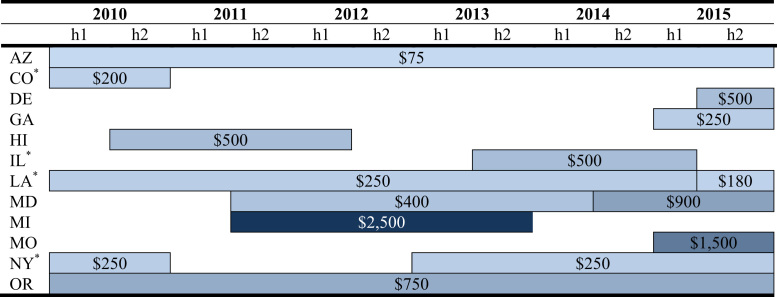
Amount of home charge subsidy. ^*^Incentive is a percentage of the cost of the equipment (CO - 20%; IL - 50%; LA - 50%, 36%; NY - 50%). Estimated amounts are based on $500 for charging equipment and $500 for installation.

**Fig. 5:**
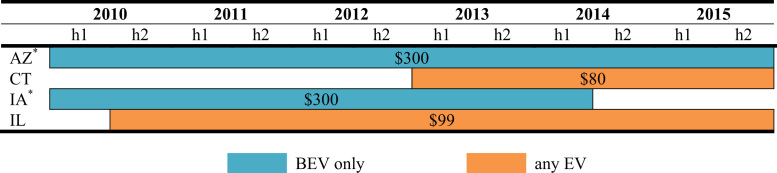
Annual value of reduced vehicle license tax or registration fee. ^*^Based on 1 percent of an MSRP of $30,000. For Arizona, the vehicle license tax amount shown reflects the amount in year one. For Iowa, the registration fee reflects only the MSRP portion in year one.

**Fig. 6:**
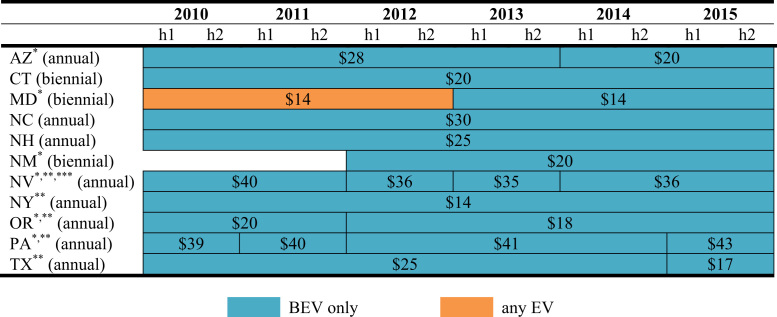
Annual value of emissions inspection exemption. ^*^County-level policy. ^**^Fees vary by area and are weighted by the area׳s population (except for PA). ^***^EREVs are exempt.

**Fig. 7:**
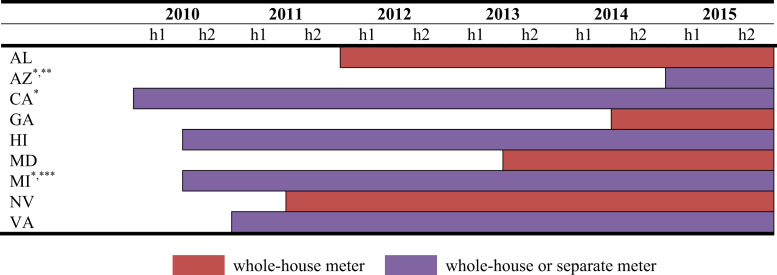
TOU electricity rates. ^*^Offered by more than one utility. ^**^Arizona Public Service Company׳s rate applies to the whole house and the Salt River Project׳s rate is designed for a separate meter. ^***^DTE Electric Company specifically requires a separate meter while Consumers Energy provides both options.

**Fig. 8:**
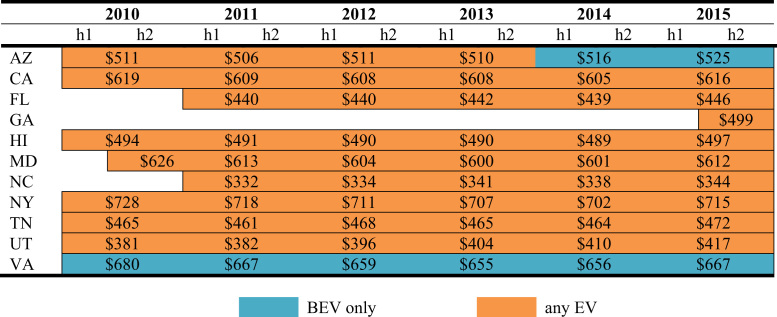
Annual value of HOV lane access.

**Fig. 9:**
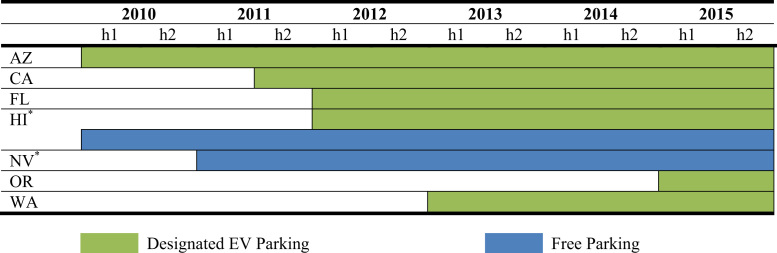
Designated or free parking.

**Fig. 10:**
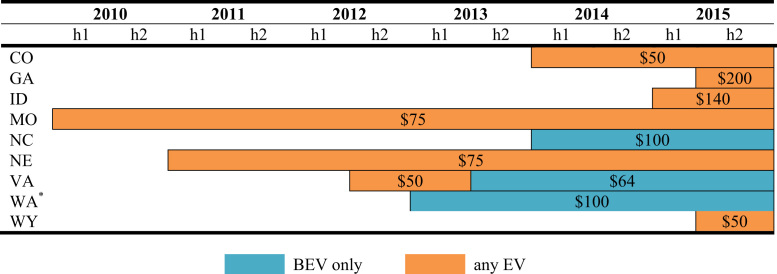
Annual EV fees ^*^Also applies to EREVs.

The data is used in Wee et al. [1] and consists of nine policy instruments—*vehicle purchase incentives*, *home charger subsidies*, *vehicle license tax* or *registration fees*, *annual EV fee, emissions inspection exemptions* and *high occupancy vehicle (HOV) lane access*, *time-of-use (TOU) rates* for electricity, *designated parking* and *free parking*. As shown in Fig. 1, Fig. 2, Fig. 3, Fig. 4, Fig. 5, Fig. 6, Fig. 7, Fig. 8, Fig. 9, Fig. 10, policy instruments are differentiated by vehicle type (BEV and PHEV) and shaded when in effect for each semi-annual period. Where applicable, amounts are also included in nominal terms for each time period.

### 1.1 Capital financial incentives

#### 1.1.1 EV purchase incentive

A total of nineteen states had an EV purchase incentive between 2010 and 2015. The timing for state rebates, income tax credits, excise tax credits and sales tax exemptions, is shown in Fig. 1. The maximum value of state EV purchase incentives is shown in Fig. 2. In many cases, the value of policies is specific to EV models because of they either depend on battery size or MSRP. Thirty-two models were considered within this dataset that were sold within the U.S. between 2010 and 2015: BMW ActiveE, BMW i3, BMW i3REx, BMW i8, BMW X5, Cadillac ELR, Chevrolet Spark, Chevrolet Volt, FIAT 500, Fisker Karma, Ford C-Max, Ford Focus, Ford Fusion, Honda Accord, Honda Fit, Hyundai Sonata, Kia Soul, McLaren P1, Mercedes-Benz B-Class, Mitsubishi i-MiEV, Nissan Leaf, Porsche 918, Porsche Cayenne, Porsche Panamera, Smartcar Fortwo, Tesla Model S, Tesla Model X, Tesla Roadster, Toyota Prius Plug-In, Toyota RAV4, Toyota Scion, Volkswagen Golf. Within all figures, the notation “h1” and “h2” means the first or second half of the year.

EV purchase incentives are provided in a variety of forms: a fixed subsidy, a percentage of the vehicle cost that may be coupled with a cap at a certain cost, and a subsidy tied to battery capacity, or some combination of battery capacity and vehicle cost. For purchase incentives based on EV cost, the manufacturer suggested retail prices (MSRP) provided by the AFDC [2] and Fueleconomy.gov [3] are used. States vary their purchase incentives by EV type and over time. For example, from 2010 Utah provided a $605–750 tax credit for both PHEVs and BEVs and in 2015 implemented an up to $1500 tax credit for BEVs and an up to $1000 tax credit for PHEVs.

In some states, programs providing EV purchase subsidies sunset on a specified date. In others, programs continue until annual or total set-aside funds are exhausted. For example, in South Carolina, total claims by taxpayers cannot exceed $200,000 in a single year. In Texas, allowances end with the sunset date or when funds allotted to the program are exhausted. For a few states, Hawaii and Oregon, subsidies only went to pre-2013 EV adopters. In states such as Vermont, program eligibility was limited to a fixed number of customers.

Two states, New Jersey and Washington, have a sales tax exemption. New Jersey׳s sales tax exemption was enacted in 2004 and applies only to BEVs. Washington׳s tax exemption began in 2009 and was extended in July 2015 from only BEVs and other zero emission vehicles to PHEVs capable of travelling 30 miles using only battery power (known as extended range electric vehicles, EREVs). To be eligible for the sales tax exemption, vehicles must be valued at $35,000 or less.

#### 1.1.2 Home charger subsidy

Ten states have offered incentives for EV owners to install Level 2 home charging stations. Level 1 chargers are included with the vehicle and plug into the standard household outlet; Level 2 home chargers are installed on a 240-volt dedicated circuit to enable faster charging. Subsidies for Level 2 charging hardware and/or installation are offered by state governments and some utility companies. Fig. 3, Fig. 4 display the duration and value of the home charging incentive by the type of subsidy and type of expenditure subsidized, where “C” denotes charger and “I” installation.

States configure their home charger subsidies based either on fixed amounts or as a percentage of the equipment׳s price and/or installation costs. Amongst the home charger policies implemented at the state-level, Missouri has the most generous incentive, up to $1,500 for hardware and installation, while Arizona has the least generous, providing a maximum of $75 for installation. Colorado offers a 20% subsidy while Illinois, New York, and Louisiana (in its first iteration of this policy) offer a 50% subsidy. Maryland originally offered up to a $400 income tax credit for the cost of the hardware, and then converted it to a rebate capped at $900 that covers both the hardware and installation cost. Similar to vehicle purchase incentives, the durations of home charger subsidy programs are subject to funding caps and sunset dates.

Utilities servicing the majority of residential customers in two states, Georgia and Michigan, also provide a subsidy for home chargers [4]. Some utilities have provided free or subsidized equipment and/or installation as part of pilot programs to gather information on EV charging habits and grid impacts.

### 1.2 Operating financial incentives

#### 1.2.1 Reduced vehicle license tax or registration fees

Arizona has a reduced vehicle license tax (VLT) for EVs while Connecticut, Illinois, Iowa provide a reduced registration fee for EVs. The value of the policy is calculated with respect to the fee that would have been paid absent the policy. For illustration, Fig. 5 displays the annual value and duration of these policies. For Connecticut, vehicles are charged $80 every two years while EVs pay $38 every two years. For Illinois, conventional vehicles pay $99 annually while EVs pay $35 every two years. In Iowa, the registration fee for a BEVs is $25 while all other vehicles are assessed based on MSRP according to the vehicle age plus a nominal fee based on vehicle weight. In Arizona, the VLT for conventional vehicles is 1 percent of the MSRP in its initial year, with the VLT cut by 15 percent in each subsequent year. The value of the reduced VLT and registration fee are calculated for Arizona and Iowa, respectively, at the model level based on MSRP.

#### 1.2.2 Emissions inspection exemptions

The Federal Clean Air Act (42 U.S.C. § 7511a) mandates emission testing in major metropolitan areas that do not meet ambient air quality standards. Among regulated states,^1^ some do not require or waive emission inspection requirements for EVs. Fig. 6 displays states that exempt EVs from emissions testing and the estimated annual savings from the exemption. Because fees in some states vary by area (county or metropolitan area), they are aggregated and weighted by area population. In Pennsylvania, fees vary by county and station; an unweighted average of station fees for each county provided by the State of Pennsylvania is used.

#### 1.2.3 Time-of-Use (TOU) electricity rates

Many utilities offer EV-oriented TOU electricity rates, often as a pilot program, to encourage off-peak charging. Eligibility for TOU rates may require a separate meter for EV charging or EV charging can be combined with household consumption. The states presented in Fig. 7 have TOU rates offered specifically for EVs by utilities which service over 50 percent of residential customers in the state [4]. TOU programs are offered by the following utilities: Alabama (Alabama Power), Arizona (Arizona Public Service Company, Salt River Project), California (Pacific Gas & Electric, Los Angeles Water Department and Power, Sacramento Municipal Utility District, San Diego Gas and Electric, Southern California Edison), Georgia (Georgia Power), Hawaii (Hawaiian Electric Companies), Maryland (Baltimore Gas & Electric), Michigan (DTE Electric Company, Consumers Energy), Nevada (NV Energy), and Virginia (Virginia Dominion Power).

### 1.3 Preferred access incentives

#### 1.3.1 HOV lane exemption

Fig. 8 displays eleven states with HOV lane exemptions for EVs regardless of their passenger count and shows the estimated annual value of access to an HOV lane for an EV driver [5].

Some states, like California, require EVs to have special decals or license plates to provide visible identification when they are driven in HOV lanes. Some states charge a minimal fee and a few states, such as Arizona, California, and Utah, only issue a limited number of permits.^2^ However, only Arizona hit its cap for PHEV permits within our sample period.

To estimate the value of HOV lane access, a measure of congestion in major U.S. metropolitan areas from 1982 to 2014 provided by the Texas A&M Transportation Institute׳s (TTI) Urban Mobility Scorecard [6] is used as a starting point. Using Bento et al.’s [7] estimate for the value of HOV lane access in Southern California ($743 annually) divided by TTI׳s estimate of congestion for the Los Angeles area in the same year, yields a parameter that reflects the percent of congestion costs that could be relieved by access to HOV lanes, 43 percent in Southern California. The state׳s level of congestion is estimated by taking a population weighted average of metropolitan area congestion for the state. The share parameter is applied to estimate the value of HOV lane access per person by state from 2010 to 2014 [8]. Missing 2015 values are generated by increasing the 2014 values by the change in the U.S. All Urban Consumers consumer price index (CPI) [9]. Using this methodology, the annual value of HOV lane access for EVs ranges between $300 and $700. Note that the application of parameters derived from Southern California to the other 49 states is a crude approximation for the value of HOV lane access in those states. Moreover, even within California, this value does not accrue to all EV owners, as proximity to and use of HOV lanes vary widely.

#### 1.3.2 Designated parking and free parking

Six states mandate designated parking for EVs, as shown in Fig. 9. Hawaii goes further by requiring all parking lots with more than 100 stalls to provide an EV space with charging facilities. Two states, Hawaii and Nevada,^3^ offer free parking in public metered areas, also shown in Fig. 9. Hawaii is the only state where free parking is also available at state airports.

### 1.4 Annual EV Fee

A growing number of states are imposing an annual fee on EVs to make up for lost gasoline taxes. This fee is assessed in addition to the standard registration fee. Fig. 10 displays the duration and amounts of the annual fee, which range from $50 to $200.

## 2 Experimental design, materials and methods

The Alternative Fuel Database Center (AFDC) was our baseline resource to document existing and expired state policies related to EVs [10]. However, the timing and duration of state policies were often missing. In addition, sub-state level policies are documented to the extent that they affected a majority of the state׳s population or customer base. Publicly available data within the AFDC is supplemented with information from over 300 government (state, city, and county) and utility websites, including use of the WayBackMachine [11], a website archive. Because most government websites have up-to-date information on current policies, they often lack information on introduction dates and policies that have since expired. The use of this website archive allowed for filling in data from earlier versions of state and local government websites. For missing information unavailable online, phone and email inquiries were made to various state departments (Taxation, Revenue, Transportation, Motor Vehicles, Energy, Environmental Quality, Public Utilities Commission, etc.) and Clean Cities Coalitions, a state and city-level organization supported by the U.S. Department of Energy. A total of nine policy instruments are documented, including the timing of their introduction and, where relevant, end date. Though there may be instances where legislation has passed and had a substantial lag in implementation, to the best of our ability, the semi-annual dates reported reflect dates of policy implementation. In addition to the types and timing of policy instruments, their estimated value is also provided when possible. For *vehicle purchase incentives*, *home charger subsidies*, *vehicle license tax* or *registration fees*, and the *annual EV fee*, these are documented in current dollars. For *emissions inspection exemptions* and *high occupancy vehicle lane access*, assumptions are made to estimate the annual value of these policy instruments. Note, the data presented in Fig. 1, Fig. 2, Fig. 3, Fig. 4, Fig. 5, Fig. 6, Fig. 7, Fig. 8, Fig. 9, Fig. 10 aggregate the detailed data collected by model within each state and semi-annual time period. The disaggregated data can be downloaded in csv format here: https://sites.google.com/a/hawaii.edu/sherilyn/statepoliciesbymodel.csv?attredirects=0&d=1.

## Footnotes

1 A state is considered to have an emission inspection policy if the population of the affected counties comprise over 50 percent of the population in the state, where population is taken from the U.S. Census Bureau [5].

2 Permit fees were not taken into account in our calculations of the value of HOV lane access.

3 State legislation mandates that local jurisdictions create free parking programs for AFVs in Nevada. The City of Reno and City of Nevada have established programs.

## References

[1] Wee S., Coffman M., Croix S. La (2018). Do electric vehicle incentives matter? Evidence from the 50 U.S. States. Res. Policy.

[2] Alternative Fuels Data Center (AFDC), Vehicle Cost Calculator, 2017. 〈https://www.afdc.energy.gov/calc/〉, (Accessed on 18 July 2017).

[3] Fueleconomy.gov, Compare Side-by-Side, U.S. Department of Energy, 2018. 〈https://www.fueleconomy.gov/〉, (Accessed on 26 April 2018).

[4] U.S. Energy Information Administration (EIA), Electric power sales, revenue, and energy efficiency. Form EIA-861 detailed data files, U.S. Department of Energy (DOE), 2011–2016. 〈https://www.eia.gov/electricity/data/eia861/〉, (Accessed on 14 April 2016).

[5] Alternative Fuels Data Center (AFDC), High Occupancy Vehicle (HOV) Lane Exemption, U.S. Department of Energy, 2016. (Accessed on 1 September 2016 at 〈http://www.afdc.energy.gov/laws/386〉).

[6] Texas A&M Transportation Institute, Urban Mobility Information: 2015 Urban Mobility Scorecard, 2015. 〈https://mobility.tamu.edu/ums/〉, (Accessed on 26 April 2018).

[7] Bento A., Kaffine D., Roth K., Zaragoza-Watkins M. (2014). The effects of regulation in the presence of multiple unpriced externalities: evidence from the transportation sector. Am. Econ. J.: Econ. Policy.

[8] U.S. Census Bureau, Annual Estimates of the Resident Population: April 1, 2010 - July 1, 2015.

[9] Bureau U.S. (2016). of Labor Statistics (BLS), Consumer Price Index - All Urban Consumers.

[10] Alternative Fuels Database Center (AFDC), State Laws and Incentives, U.S. Department of Energy, 2016. 〈http://www.afdc.energy.gov/laws/state〉, (Accessed on 15 July 2016).

[11] Internet Archive, WayBackMachine, 2018. 〈https://web.archive.org〉, (Accessed on 22 March 2018).

